# Pseudo-acetylation of K326 and K328 of actin disrupts *Drosophila melanogaster* indirect flight muscle structure and performance

**DOI:** 10.3389/fphys.2015.00116

**Published:** 2015-04-28

**Authors:** Meera C. Viswanathan, Anna C. Blice-Baum, William Schmidt, D. Brian Foster, Anthony Cammarato

**Affiliations:** Division of Cardiology, Department of Medicine, Johns Hopkins University School of MedicineBaltimore, MD, USA

**Keywords:** tropomyosin, myosin, acetylation, muscle contraction, post-translational modification

## Abstract

In striated muscle tropomyosin (Tm) extends along the length of F-actin-containing thin filaments. Its location governs access of myosin binding sites on actin and, hence, force production. Intermolecular electrostatic associations are believed to mediate critical interactions between the proteins. For example, actin residues K326, K328, and R147 were predicted to establish contacts with E181 of Tm. Moreover, K328 also potentially forms direct interactions with E286 of myosin when the motor is strongly bound. Recently, LC-MS/MS analysis of the cardiac acetyl-lysine proteome revealed K326 and K328 of actin were acetylated, a post-translational modification (PTM) that masks the residues' inherent positive charges. Here, we tested the hypothesis that by removing the vital actin charges at residues 326 and 328, the PTM would perturb Tm positioning and/or strong myosin binding as manifested by altered skeletal muscle function and structure in the *Drosophila melanogaster* model system. Transgenic flies were created that permit tissue-specific expression of K326Q, K328Q, or K326Q/K328Q acetyl-mimetic actin and of wild-type actin via the UAS-GAL4 bipartite expression system. Compared to wild-type actin, muscle-restricted expression of mutant actin had a dose-dependent effect on flight ability. Moreover, excessive K328Q and K326Q/K328Q actin overexpression induced indirect flight muscle degeneration, a phenotype consistent with hypercontraction observed in other *Drosophila* myofibrillar mutants. Based on F-actin-Tm and F-actin-Tm-myosin models and on our physiological data, we conclude that acetylating K326 and K328 of actin alters electrostatic associations with Tm and/or myosin and thereby augments contractile properties. Our findings highlight the utility of *Drosophila* as a model that permits efficient targeted design and assessment of molecular and tissue-specific responses to muscle protein modifications, *in vivo*.

## Introduction

Striated muscle contraction results from transient interactions between myosin-containing thick filaments and actin-containing thin filaments. Contractile regulation is achieved by Ca^2+^-dependent modulation of myosin S1 cross-bridge binding to actin by the thin filament-associated troponin-tropomyosin complex (reviewed in Tobacman, 1996; Gordon et al., 2000; Brown and Cohen, 2005; Lehman and Craig, 2008). The location of continuous troponin-tropomyosin complexes along the surface of F-actin governs the access of myosin binding sites and, hence, force production (Haselgrove, 1973; Huxley, 1973; Parry and Squire, 1973; McKillop and Geeves, 1993; Lehman et al., 1994; Vibert et al., 1997). Under conditions of low Ca^2+^, the troponin complex constrains tropomyosin (Tm) in a position that occludes myosin target sites on actin. Consequently, Tm sterically blocks and limits myosin binding, and relaxation results. During muscle activation, Ca^2+^ binds to troponin and triggers movement of Tm away from myosin binding sites. This relocation partially relieves the structural blocking imposed by Tm. Initial myosin binding on thin filaments further displaces Tm and exposes myosin binding sites along F-actin, thereby contributing to the cooperative activation of contraction.

**Figure 1:**
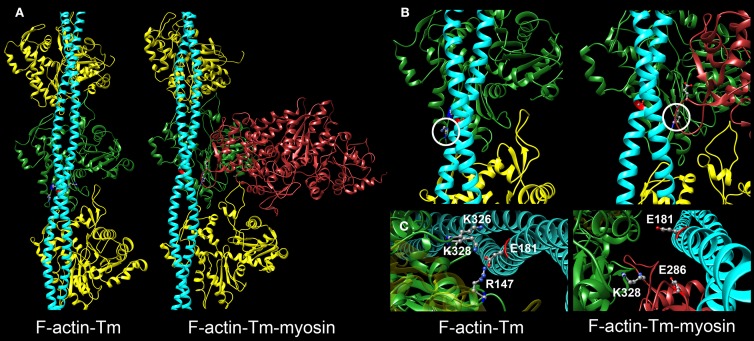
**Critical electrostatic F-actin-Tm and F-actin-Tm-myosin interactions. (A)** Molecular models showing the location of tropomyosin (Tm) (blue) on actin (yellow and green) in the absence and presence of the myosin head (S1) (red) bound in rigor. The F-actin-Tm and rigor F-actin-Tm-myosin structures are based on those generated by Li et al. (2011) and Behrmann et al. (2012) respectively. **(B)** Enlarged views illustrate critical electrostatic associations between actin and Tm in the absence or presence of S1. K328 on actin (circled) interacts with E181 of Tm in the absence of myosin (left) and with E286 of myosin when S1 is bound in rigor (right). Note the azimuthal movement of Tm across F-actin. **(C)** Projected and enlarged views highlight vital electrostatic interactions of actin residues R147, K326, and K328 with E181 of Tm, in the absence of myosin, and of K328 of actin and E286 of S1 when myosin is bound in rigor. These associations are likely critical for thin filament and muscle function.

Although the specific residues that constitute the binding interface of actin and Tm are not completely known, it is well accepted that the association of Tm with actin is largely electrostatic (Lorenz et al., 1995; Brown and Cohen, 2005; Barua et al., 2011, 2013; Li et al., 2011). Recently, models of the conserved binding interface between actin and Tm in various states have been proposed based on molecular evolutionary and mutational analysis, computational chemistry, and electron microscopy reconstructions (Barua et al., 2011, 2013; Li et al., 2011; Behrmann et al., 2012; Von Der Ecken et al., 2015). Structural studies of F-actin-Tm and F-actin-Tm-myosin revealed that several amino acids on actin can potentially form distinct contacts with Tm in the absence and presence of myosin S1 (Li et al., 2011; Behrmann et al., 2012; Von Der Ecken et al., 2015). For example, in the absence of S1, a cluster of basic actin residues comprised of K326, K328, and R147 appeared poised to clasp onto E181 of Tm to establish highly favorable associations (Figure 1) (Li et al., 2011). Interestingly, K328 also interacts electrostatically with E286 of S1 to help define a strong contact point between actin and rigor-bound myosin (Behrmann et al., 2012).

Alterations to thin filament proteins affect the properties of muscle. Missense mutations, for instance, can disrupt conserved interfaces among cardiac thin filament subunits and initiate diverse cardiomyopathies (Tardiff, 2011). Similar to disease-causing mutations, post translational modifications (PTMs) also alter the chemical nature of thin filament subunits. Although less-well appreciated, these modifications are widely employed *in vivo*, occur through enzymatic and non-enzymatic mechanisms, and direct both physiological and pathological processes (Terman and Kashina, 2013). PTMs add or remove a functional group to or from specific amino acid residues, which can induce changes in protein structure, activity, or binding partners (van Eyk, 2011). To date, more than 400 different PTMs have been described, although far fewer have been documented in higher organisms (Agnetti et al., 2011). In the cardiac subproteome, phosphorylation is by far the best-described PTM (Sumandea et al., 2004; Agnetti et al., 2011; Solaro and Kobayashi, 2011; van Eyk, 2011). It has been observed for 80% of the myofilamentous proteins (Agnetti et al., 2011). However, the effects of the majority of the potential PTMs have not been fully investigated, in part because of a lack of technologies needed to target and reliably identify them. Moreover, compared to inherited mutations, the influence of relatively few PTMs on contractile performance have been examined in the physiological context of muscle.

**Figure 2:**
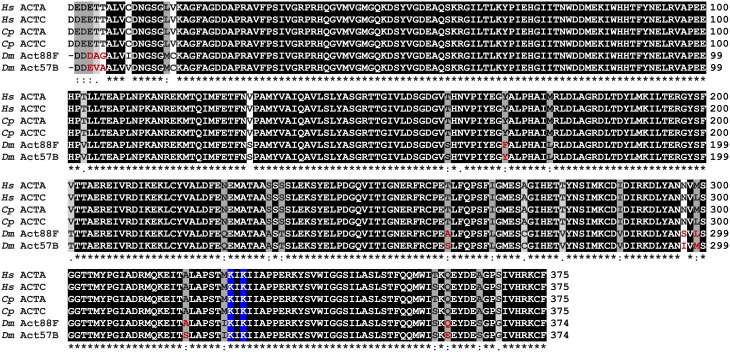
**Multiple sequence alignment of actin isoforms**. Multiple sequence alignment of skeletal and cardiac actin from *Homo sapiens* (*Hs*), *Cavia porcellus* (*Cp*), and *Drosophila melanogaster* (*Dm*) reveals highly conserved proteins. ACTA, skeletal muscle actin; ACTC, cardiac muscle actin; Act88F, *Drosophila* indirect flight muscle actin; Act57B, *Drosophila* cardiac actin. Residues are shaded based on degree of conservation. An (^*^) indicates positions that have identical residues, a (:) indicates substitution with high structural similarity, and a (.) indicates substitution low structural similarity.

Actin is an abundant and highly conserved protein (Figure 2). It participates in more protein-protein interactions than any known protein and is subject to a number of PTMs (Herman, 1993; Dominguez and Holmes, 2011; Terman and Kashina, 2013). Characterizing these modifications constitutes a rapidly expanding area within actin studies and muscle biology (Terman and Kashina, 2013). Phosphorylation, methylation, ADP-ribosylation, oxidation, arginylation, O-GlcNAcylation, ubiquitylation, and acetylation are examples of major actin modifications, many of which have now been confirmed in sarcomeric actin. For example, K326 and K328 of actin isolated from various cell lines were shown to be acetylated (Choudhary et al., 2009) and, as recently described by Foster et al. (2013) these residues were also shown to be acetylated on actin recovered from the myofilament fraction of guinea pig hearts. Acetylation is a reversible PTM that neutralizes the positive charge of K326 and K328 on actin. Unlike the N-terminal acetylation, acetylation of these residues has unknown function. Based on *in vitro* and *in silico* structural predictions, removal of positively charged amino acids that are potentially critical to Tm and myosin electrostatic associations likely alters muscle performance substantially and in diverse ways.

Here, we aimed to investigate the *in vivo* physiological and morphological consequences of acetylating actin residues K326 and K328 on muscle. We chose the indirect flight muscle (IFM) of *D. melanogaster* as our primary experimental vehicle. *Drosophila* IFMs are highly amenable to mechanical and structural analyses, are not required for viability, exhibit a stretch activation response that is similar to that of cardiac muscle, and provide ample material that is easily isolated for biochemical and biophysical experimentation (Bing et al., 1998; Razzaq et al., 1999; Cammarato et al., 2004; Vikhorev et al., 2010; Swank, 2012). The vast array of genetic tools available to manipulate the fly's genome also provides unique opportunities to examine how thin filament modifications affect muscle structure and performance. The GAL4-UAS system simplifies tissue-specific expression of mutant transgenes (Brand and Perrimon, 1993). However, previous studies suggested that IFM myofibril assembly and flight ability are highly sensitive to the stoichiometry of muscle proteins (Beall and Fyrberg, 1991; Bernstein et al., 1993), and therefore, the utility of this bipartite expression system for investigating major contractile components has been questioned. For example, despite normal sarcomere appearance, overexpression of several *UAS-GFP-actin* alleles was shown to exclusively affect IFM function, leading to flightless adults that were otherwise healthy (Röper et al., 2005). The effects of non-GFP fusion constructs on IFM performance were not tested. Therefore, to determine if the IFM can serve as a viable, conditional model system for probing the effects of PTMs on myofibrillar components, we tested the hypotheses that (1) GAL4-UAS-mediated overexpression of a *Drosophila* cardiac actin isoform in the IFM would not perturb gross morphology or flight performance as determined by standard metrics and that (2) expression of K326Q, K328Q, or K326Q/K328Q acetyl-mimetic cardiac actin disrupts vital electrostatic interactions required for Tm positioning and/or strong myosin binding, *in vivo*, as manifested by perturbed IFM structure and function. Using four muscle-specific GAL4 drivers we found that expressing wild-type *Drosophila* cardiac actin had no effect on flight or gross muscle organization. Relatively high expression levels of K326Q cardiac actin mildly affected flight ability, whereas excessive amounts of K328Q and K326Q/K328Q cardiac actin eliminated flight and triggered IFM destruction. Based on F-actin-Tm and F-actin-Tm-myosin models and on our physiological data, we propose that acetylating K326 and K328 of actin alters crucial electrostatic associations with Tm and/or myosin and thereby promotes actomyosin associations and modulates muscle performance. Our findings highlight the utility of *Drosophila* as a model that permits efficient targeted design and assessment of tissue-specific responses to muscle protein modifications, *in vivo*.

## Materials and methods

### Structural modeling

Models of the F-actin-Tm (Li et al., 2011; Orzechowski et al., 2014) and the rigor F-actin-Tm-myosin (PDB ID: 4A7F) (Behrmann et al., 2012) binding interfaces were generated using the molecular modeling program, Chimera version 1.9 (Pettersen et al., 2004).

### Multiple sequence alignment

Sequence comparison of skeletal and cardiac actin isoforms from *Homo sapiens*, *Cavia porcellus*, and *D. melanogaster* was performed using the Clustal Omega multiple sequence alignment program. Residues were shaded based on degree of conservation.

### Fly stocks

All flies were raised at 25°C on a standard cornmeal-yeast-sucrose-agar medium. The *w*^*1118*^ strain was obtained from Genetic Services Inc. (Sudbury, MA) and the *Mef2-GAL4* driver line (*y*^*1*^ w^*^; *P{GAL4-Mef2.R}3*) from the Bloomington *Drosophila* Stock Center (Bloomington, IN). The *MHC-GAL4* (*MHC-GAL4*^*82*^) line described by Marek et al. (2000) was obtained from Dr. Rolf Bodmer (Sanford Burnham Medical Research Institute, La, Jolla, CA). The *UH3-GAL4* (Singh et al., 2014) and the *Act88F-GAL4* (88F2) (Bryantsev et al., 2012) lines were gifts from Dr. Upendra Nongthomba (Indian Institute of Science, Banglore, India) and Dr. Richard M. Cripps (University of New Mexico, Albuquerque, NM) respectively. The *Mhc*^*10*^ (*w*; *Mhc*^*10*^/*Mhc*^*10*^; *TM2/MKRS*) IFM-specific myosin null line was acquired from Dr. Sanford I. Bernstein (San Diego State University, San Diego, CA). *Mef2-GAL4*> *UAS-Act57B*^*WT*^; *Mhc*^*10*^/+ and *UAS-Act57B*^*K*328*Q*^; *Mhc*^*10*^/+ *Drosophila* were generated by standard mating procedures.

### Construction of *UAS-Actin* transgenes

The N-terminally-labeled GFP-actin construct (*pUASp.Act57B*^*GFP.WT*^) was generously provided by Dr. Katja Röper (MRC- Laboratory of Molecular Biology, Cambridge, UK). The *Act57B*^*GFP.WT*^ and *Act57B*^*WT*^ cDNA sequences were subsequently inserted into the pUASTattB vector (obtained from Dr. Christopher Potter, Johns Hopkins University) using the KpnI and XbaI and the NotI and XbaI restriction sites respectively. The pUASTattB vector includes the *Drosophila miniwhite* (*w*^*+*^) gene as a selectable eye color marker. The Act57B actin acetyl-mimetic mutations, K326Q, K328Q, and K326Q/K328Q, were generated by site-directed mutagenesis using specific primer pairs and the QuikChange Site-directed mutagenesis kit (Agilent Technologies).

List of primers for site-directed mutagenesis:

**Table:** 

Act57B^K326Q^ (+) primer	5′ CCATCCACCATCCAGA TCAAGATCATT 3′
Act57B^K326Q^ (−) primer	5′ AATGATCTTGATCTGG ATGGTGGATGG 3′
Act57B^K328Q^ (+) primer	5′ ACCATCAAGATCCAGA TCATTGCTCCC 3′
Act57B^K328Q^ (−) primer	5′ GGGAGCAATGATCTGG ATCTTGATGGT 3′
Act57B^K326Q/K328Q^ (+) primer	5′ TCCACCATCCAGATCC AGATCATTGCT 3′
Act57B^K326Q/K328Q^ (−) primer	5′ AGCAATGATCTGGAT CTGGATGGTGGA 3′

### Generation of transgenic *Drosophila*

The pUASTattB constructs were injected into attp40 *Drosophila* embryos for PhiC31 integrase mediated site-specific transgenesis (transgene landing site cytolocation 25C7) by Genetic Services, Inc. Injected adults were crossed to *w*^*1118*^ flies and the progeny screened for pigmented eye color, which reflects the presence of the *miniwhite* (*w*^+^) marker and the transgene, an indicator of successful transformation. Each transformant fly was then crossed into the *w*^*1118*^ background to generate stable transgenic lines.

### Verification of transgene expression

Transgenic actin expression was verified by isolating total RNA from bisected thoraces or dissected IFMs of 10 *Mef2-GAL4*> *UAS-Act57B*^*WT*^, *UAS-Act57B*^*K326Q*^, *UAS-Act57B*^*K328Q*^, or *UAS-Act57B*^*K326Q/K328Q*^ flies using the Quick-RNA microprep kit (Zymo Research Corp). Contaminating DNA was removed with RNase free DNase I (Zymo Research Corp). One step RT-PCR was carried out with the Qiagen QuantiTect Reverse Transcription Kit (Qiagen Inc) and 10 ng RNA per reaction. The cDNA was amplified using an *Act57B* primer pair (5′ CCCTGTACGCCTCCGGTCGTA 3′ and 5′ TTAGAAGCACTTGCGGTGGAC 3′) and the amplified product was sequenced at the Johns Hopkins Synthesis and Sequencing facility.

Transgenic muscle-restricted protein expression was confirmed using the *UAS-Act57B*^*GFP.WT*^ reporter line in conjunction with either the *MHC-GAL4* or *Mef2-GAL4* drivers. Two-day-old adult progeny were imaged using a Leica M165FC fluorescent stereo microscope and a Leica EC3 digital camera.

### Protein quantification

Virgin *MHC*-, *Mef2*-, *UH3*-, and *Act88F-GAL4* female flies were crossed with male flies harboring the *UAS-Act57B*^*GFP.WT*^ transgene. To quantitate the amount of Act57B^GFP.WT^ transgenic actin driven by each muscle-specific driver, two whole thoraces of, or IFMs from three resulting progeny were dissected and homogenized in Laemmli Sample Buffer (Bio-Rad Laboratories). Each biological sample was then incubated briefly at 100°C and increasing amounts of protein from each sample were loaded on a 4–15% SDS-PAGE gel (Bio-Rad Laboratories), electrophoresed, and blotted onto a nitrocellulose membrane using the Trans-Blot® TurboTM Transfer system (Bio-Rad Laboratories). Membranes were blocked with gentle shaking in PBS odyssey blocking buffer (LI-COR Biosciences) for one hour, and incubated with primary rabbit anti-actin (Proteintech), goat anti-GFP (R&D), and goat anti-GAPDH (Genscript) antibodies overnight at 4°C. The membranes were rinsed three–four times, 10–15 min each in 1x TBST (1X TBS with 0.1% tween-20) and then probed with Donkey anti-rabbit and Donkey anti-goat IRDye secondary antibodies (LI-COR Biosciences) for 60–90 min at room temperature. The membranes were rinsed again two–three times, 10–15 min each with 1X TBST followed by a final rinse in 1X TBS. The membranes were subsequently scanned using an Odyssey Infrared Imager (LI-COR Biosciences) (λ = 700 and 800 nm) and analyzed using Odyssey Application Software (v3.030, LI-COR Biosciences). Thoracic and IFM protein quantification was performed on five or eight independent biological samples respectively, with six or four technical replicates each. Mean values (± SEM) of actin and GFP intensities normalized to respective GAPDH intensities were determined. Significance was assessed via One-Way ANOVA with a Bonferroni's multiple comparison test for thoracic, and via the Mann-Whitney test for IFM samples using GraphPad Prism5.

### Flight testing

Flight tests were performed as described by Drummond et al. (1991). Newly eclosed male and female flies were aged for two days at 25°C. Each fly was released into the center of a plexiglass chamber with a light source positioned at the top at 23°C and assigned a flight index of six for upward flight, four for horizontal, two for downward, or zero for no flight. The average flight index from 100–300 flies was calculated for each genotype. Flight assays for *Act88F-GAL4*-expressing flies were conducted exclusively on females as males consistently displayed severe flight impairment. Values represent mean ± SEM. Significance was assessed using a Kruskal-Wallis One-Way ANOVA.

### Climbing assay

Climbing tests were conducted on two-day-old flies at room temperature. Small groups of ~20 flies were placed in covered, cylindrical vials (2.5 cm diameter × 20 cm high), which were aligned with one centimeter markings to measure the height each fly climbed in five seconds. The test was repeated 10 times for each set of flies. The average climbing distance for each fly was recorded for 30–160 flies per genotype. Values represent mean ± SEM. Significance was assessed using a Kruskal-Wallis One-Way ANOVA.

### Imaging of indirect flight muscles

Polarized light microscopy of hemi-thoraces to examine the gross morphology of two-day-old *Mef2-GAL4*> *UAS-Act57B*^*WT*^, *UAS-Act57B*^*K326Q*^, *UAS-Act57B*^*K328Q*^, or *UAS-Act57B*^*K326Q/K328Q*^ adult *Drosophila* IFM was performed as described previously (Nongthomba and Ramachandra, 1999). Briefly, flies were anesthetized, and heads and abdomens removed. Thoraces were fixed overnight in 4% formaldehyde at 4°C and rinsed in PBS the following day. The specimens were laid supine on a glass slide and snap frozen in liquid nitrogen for 10 s. Frozen thoraces were immediately bisected down the midsagittal plane using a razor blade and IFMs were visualized using a Leica DM5000B microscope at 10X magnification with polarizing filters. Images were taken with a Hamamatsu digital camera.

Fluorescent microscopy was employed to improve pathohistological characterization of IFMs from young (< four hour old) and two-day-old adult *Act88F-GAL4*> *UAS-Act57B*^*WT*^, *UAS-Act57B*^*K326Q*^, *UAS-Act57B*^*K328Q*^, and *UAS-Act57B*^*K326Q/K328Q*^ *Drosophila*. Thoraces were prepared and bisected as described above, followed by staining with 1:100 Alexa-594 Phalloidin in PBST overnight at 4°C. Samples were rinsed with PBS before imaging with the EVOS® FL Cell Imaging System (Life Technologies) at 4X magnification.

## Results

### Actin sequence analysis

The genomes of human, guinea pig, and fly contain six highly conserved actin genes. As found in vertebrates, *D. melanogaster* expresses specific isoforms of actin in adult skeletal and cardiac muscle. The *Actin88F* (*Act88F*) gene encodes all sarcomeric actin of *Drosophila* IFM (Fyrberg et al., 1983; Hiromi and Hotta, 1985; Nongthomba et al., 2001) while *Actin57B* (*Act57B*) is one of two genes encoding sarcomeric actin in the adult fly heart (Cammarato et al., 2011; Shah et al., 2011). The skeletal and cardiac actin isoforms differ in only a few amino acids within and between species (Figure 2).

### Generation of transgenic *Drosophila* and confirmation of muscle-restricted transgene expression

To determine the consequences of masking the inherent charge of actin residues K326 and K328 *in vivo*, we generated multiple transgenic acetyl-mimetic lines that permitted muscle targeted gene expression. Use of the PhiC31 integrase system ensured all *UAS-Act57B* transgenes were integrated at an identical, predetermined genomic location (Groth et al., 2004). Thus, our results were directly comparable and any phenotypic differences in control vs. mutant flies could be directly attributed to neutralized lysine charges on the ectopically expressed actin.

**Figure 3:**
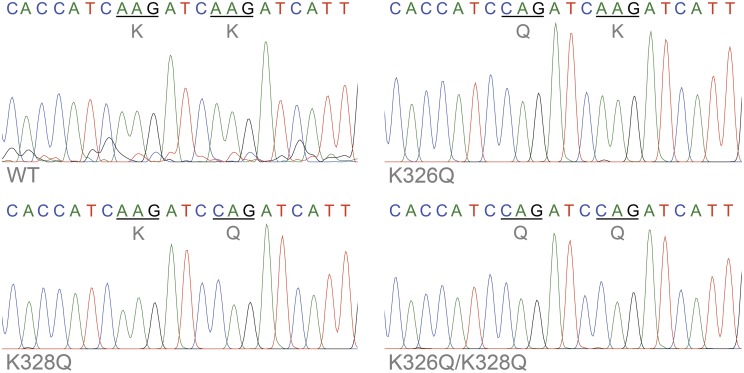
**Confirmation of transgenic actin transcription**. Sequence chromatograms of an amplified stretch of *Act57B* cDNA revealed transcription of the *UAS-Act57B* transgenes in the thoracic musculature of *Mef2-GAL4*> *UAS-Act57B* transgenic flies. The chromatograms also confirmed the presence and expression of K326Q, K328Q, or K326Q/K328Q actin mutations (identified by the AAG → CAG nucleotide transversion) in the sequenced *Act57B* cDNA fragments.

To confirm transcription of transgenic actin, flies with constructs consisting of an upstream activating sequence (UAS) followed by a downstream *Act57B*^*WT*^, *Act57B*^*K326Q*^, *Act57B*^*K328Q*^, or *Act57B*^*K326Q/K328Q*^ transgene were crossed with flies carrying the *GAL4* transactivation gene under the control of the *Mef2*-promoter (Ranganayakulu et al., 1996). The progeny inherit both genes and express the *UAS-Act57B* transgenes exclusively in musculature. Total RNA was isolated from the thoraces of young (< two days old) flies of each genotype. First-strand cDNA was synthesized followed by *Act57B* cDNA amplification. The amplified cDNA contained nucleotide sequences unique to the *Act57B* alleles, confirming transcription of the *WT*, *K326Q*, *K328Q*, or the *K326Q/K328Q Act57B* cardiac actin transgene (Figure 3). To rule out the possibility of contaminating endogenous *Act57B* cDNA originating from non-IFM thoracic musculature, amplified cDNA from the dissected IFMs of *Mef2-GAL4*> *UAS-Act57B*^*WT*^ flies was also sequenced, which verified the presence and transcription of *Act57B*^*WT*^ in *Act88F*-exclusive musculature (not shown).

**Figure 4:**
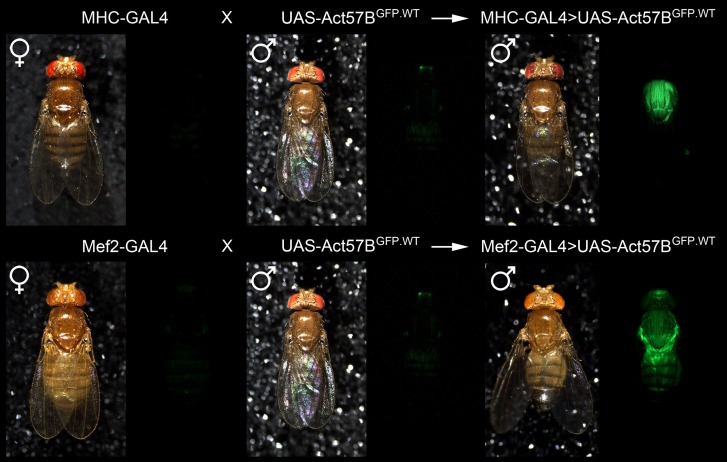
**Confirmation of muscle-restricted gene expression**. Virgin female flies expressing either the muscle-specific MHC-GAL4 or the Mef2-GAL4 driver were mated with male flies carrying the *UAS-Act57B*^*GFP.WT*^ construct. Background fluorescence coming from the musculature of the parental lines was minimal. However, fluorescence emitted from the musculature of progeny, which inherit both a GAL4 driver and the UAS-construct, was readily observed in both genotypes, confirming tissue-specific expression of transgenic Act57B actin.

To visualize muscle-restricted expression of *UAS-Act57B* transgenes, *in vivo*, flies carrying the *UAS-Act57B*^*GFP.WT*^ construct were crossed with flies harboring either the *MHC*- or *Mef2-GAL4* muscle-specific drivers. MHC-GAL4-driven transgenic Act57B^GFP.WT^ was readily observed in the thoracic musculature, which is predominantly comprised of 13 pairs of relatively large IFM fibers (Figure 4). Mef2-GAL4-driven Act57B^GFP.WT^, however, was detected far more extensively, in most somatic musculature.

### Relative to *MHC-GAL4, Mef2-GAL4* drives elevated expression levels of transgenic actin

**Figure 5:**
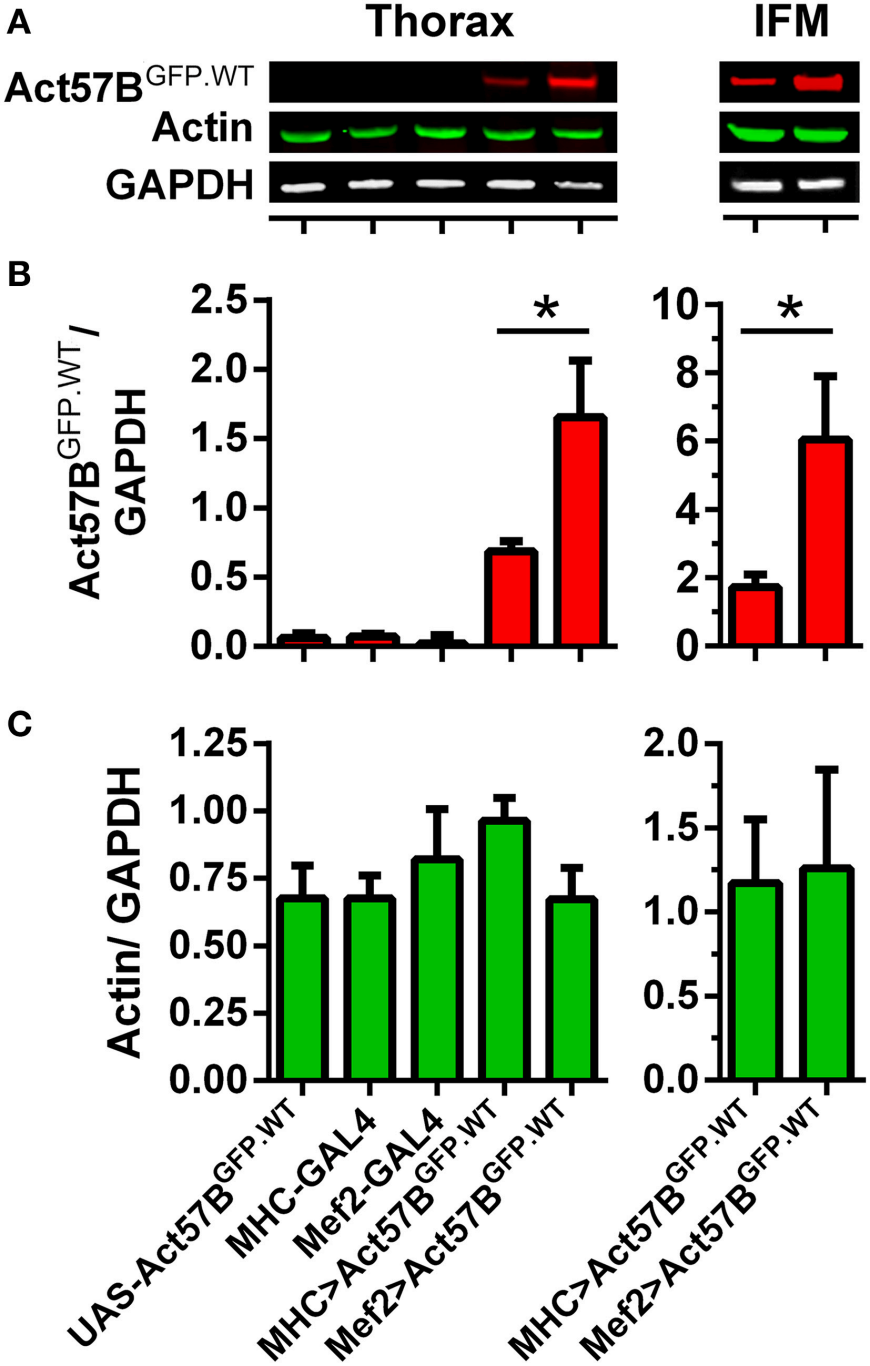
**Mef2-GAL4 drives higher expression levels of transgenic actin relative to MHC-GAL4**. Quantitative western blot analysis of steady-state Act57B^GFP.WT^ and total actin was performed on thoraces and IFMs of *Mef2-GAL4*> *UAS-Act57B*^*GFP.WT*^ and *MHC-GAL4*> *UAS-Act57B*^*GFP.WT*^ flies and of control flies two days after eclosion. **(A)** Representative western blots showing elevated thoracic (left) and IFM (right) levels of Act57B^GFP.WT^ (probed with an anti-GFP primary antibody) when driven by Mef2-GAL4 compared to MHC-GAL4. Actin and GAPDH (probed with anti-actin and anti-GAPDH antibodies) abundance appeared relatively consistent among genotypes. The GFP **(B)** and actin **(C)** intensities were measured, normalized to that of GAPDH for five thoracic samples with six technical replicates each and for eight IFM samples with four technical replicates each, and averaged for each genotype. Mef2-GAL4 drove significantly higher amounts of transgenic actin relative to MHC-GAL4 (^*^P < 0.05).

The PhiC31 integrase system for transgenic fly production limits transgene expression variability and, when used in conjunction with the GAL4-UAS system, permits the onset and magnitude of expression to be manipulated via distinct drivers (Brand and Perrimon, 1993; Groth et al., 2004; Goentoro et al., 2006). To quantify differences in transgenic protein abundance, dissected thoraces from flies expressing Act57B^GFP.WT^ actin by either MHC- or Mef2-GAL4 drivers, as well as from control “non-driven” flies, were subjected to SDS-PAGE, transferred to nitrocellulose, and probed for actin and for GFP (Figure 5A). The resulting signal intensities from GFP and actin were measured and normalized to that from GAPDH (Figures 5B,C). As expected very little GFP was distinguished among the various controls. Thoracic muscles from *MHC*- and *Mef2-GAL4*> *UAS-Act57B*^*GFP.WT*^ flies, however, exhibited detectable amounts of GFP-actin. Mef2-GAL4 induced significantly higher expression levels of the GFP-tagged actin in the thoracic musculature compared to MHC-GAL4. The resulting normalized signal was greater than two-fold higher than that determined for *MHC-GAL4*> *UAS-Act57B*^*GFP.WT*^ flies (1.66 ± 0.41 vs. 0.69 ± 0.07). These findings were corroborated using dissected IFMs, which displayed a normalized Act57B^GFP.WT^ signal that was roughly three-fold higher for *Mef2-GAL4*> *UAS-Act57B*^*GFP.WT*^ compared to *MHC-GAL4*> *UAS-Act57B*^*GFP.WT*^ fibers (6.04 ± 1.85 vs. 1.73 ± 0.37). The thoracic or IFM actin/GAPDH ratio did not differ significantly among the genotypes tested (Figure 5C). Estimation of signal intensities from the protein bands, detected exclusively with the anti-actin antibody, suggested that Mef2-GAL4> Act57B^GFP.WT^ comprises ~10–20% of total thoracic actin (not shown).

### GFP-labeled and acetyl-mimetic cardiac actin depress *Drosophila* flight ability and climbing performance

**Figure 6:**
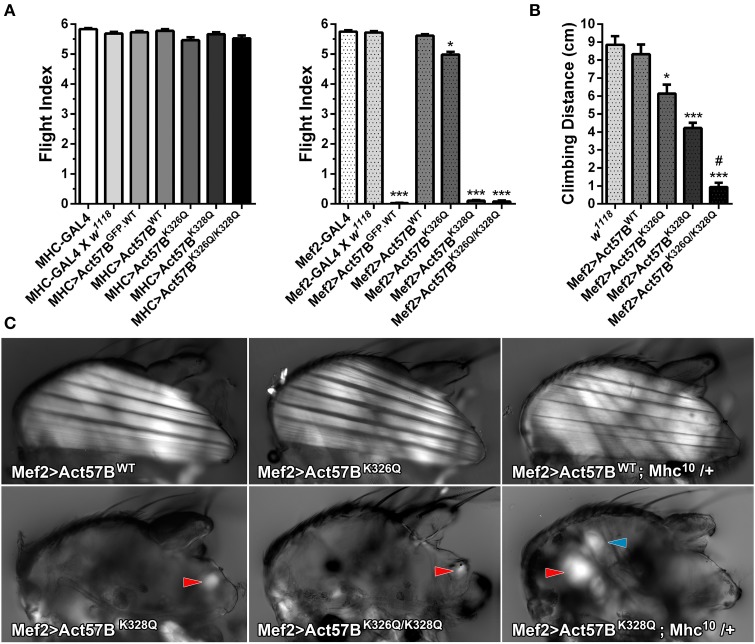
**Excessive expression levels of GFP-tagged or acetyl-mimetic actin disrupt muscle function and structure. (A)** Flight indices of control and of *MHC-GAL4*> or *Mef2-GAL4*> *UAS-Act57B*^*GFP.WT*^, *UAS-Act57B*^*WT*^, *UAS-Act57B*^*K326Q*^, *UAS-Act57B*^*K328Q*^, or *UAS-Act57B*^*K326Q/K328Q*^ transgenic *Drosophila*. MHC-GAL4 “low-dose”-driven *UAS-Act57B* constructs did not affect flight ability in any transgenic line. “High-dose” expression of *UAS-Act57B*^*GFP.WT*^ by Mef2-GAL4 abolished flight whereas *UAS-Act57B*^*WT*^ transgene expression had no effect on flight performance. Mef2-GAL4-driven expression of *UAS-Act57B*^*K326Q*^ caused a slight but significant reduction in flight ability (^*^P < 0.05 compared to controls), while expression of *UAS-Act57B*^*K328Q*^ or *UAS-Act57B*^*K326Q/K328Q*^ completely eliminated flight (^***^P < 0.001 compared to controls). **(B)** Effects of pseudo-acetylation on climbing ability. Pseudo-acetylated K326Q actin showed the least and K326Q/K328Q actin the most damaging effects, which illustrates the PTM can also influence performance of non-fibrillar muscle (^*^P < 0.05, ^***^P < 0.001 compared to controls; ^#^*P* < 0.01 compared to *Mef2-GAL4*> *UAS-Act57B*^*K328Q*^) **(C)** Polarized light micrographs of IFM from *Mef2-GAL4*> *UAS-Act57B*^*WT*^, *UAS-Act57B*^*K326Q*^, *UAS-Act57B*^*K328Q*^, or *UAS-Act57B*^*K326Q/K328Q*^ flies. *Mef2-GAL4*> *UAS-Act57B*^*K326Q*^ IFM appeared indistinguishable from *Mef2-GAL4*> *UAS-Act57B*^*WT*^ control. Mef2-GAL4-mediated expression of *UAS-Act57B*^*K328Q*^ and *UAS-Act57B*^*K326Q/K328Q*^, however, resulted in a phenotype consistent with severe hypercontraction. Minor traces of birefringent material, assumed to be IFM remnants (red arrowheads), were occasionally observed. A single copy of *Mhc*^*10*^, the IFM-specific myosin null allele, had no influence on gross muscle morphology in *Mef2-GAL4*> *UAS-Act57B*^*WT*^; *Mhc*^*10*^/+ thoraces. *Mef2-GAL4*> *UAS-Act57B*^*K328Q*^; *Mhc*^*10*^/+ *Drosophila* displayed increased abundance of birefringent thoracic musculature (red arrowhead) relative to *Mef2-GAL4*> *UAS-Act57B*^*K328Q*^ flies. The blue arrowhead indicates the tergal depressor of trochanter (jump) muscle. These findings are consistent with previous studies that demonstrated reduced MHC partially suppresses fiber destruction and they suggest that IFM expressing Mef2-GAL4-driven *UAS-Act57B*^*K328Q*^ requires relatively little myosin to hypercontract.

Flight tests were performed on two-day-old *MHC*- and *Mef2-GAL4*> *UAS-Act57B*^*GFP.WT*^, *UAS-Act57B*^*WT*^, *UAS-Act57B*^*K326Q*^, *UAS-Act57B*^*K328Q*^, or *UAS-Act57B*^*K326Q/K328Q*^ transgenic *Drosophila* lines to determine if expression of wildtype or acetyl-mimetic cardiac actin can support IFM function. The driver lines, and the progeny of each driver line crossed to *w*^*1118*^ flies, served as additional controls. *MHC*- and *Mef2-GAL4 Drosophila* by themselves, and the offspring of the driver lines crossed to *w*^*1118*^, showed normal flight indices (*FI* = 5.69–5.83) in accord with published values calculated for wildtype flies (Figure 6A) (Drummond et al., 1991; Swank et al., 2003; Suggs et al., 2007; Cammarato et al., 2008; Wang et al., 2012). “Low dose” ectopic expression of all *UAS-Act57B* actin constructs by MHC-GAL4 had no effect on flight ability. All lines performed equally well (*FI* = 5.46–5.78). Thus, MHC-GAL4-driven GFP-tagged, wildtype, or acetyl-mimetic Act57B actin supported flight.

Similar to previous results (Röper et al., 2005), *Mef2-GAL4*> *Act57B^GFP.WT^* expression eliminated flight ability (*FI* = 0.03 ± 0.01) (Figure 6A). Interestingly, relatively “high dose” expression of *Act57B* cardiac actin that lacked the GFP fusion tag had no observable effect on flight as *Mef2-GAL4*> *UAS-Act57B^WT^ Drosophila* demonstrated wildtype-like flight ability (*FI* = 5.62 ± 0.04). Unlike what was found in combination with MHC-GAL4, expression of *UAS-Act57B*^*K326Q*^ by Mef2-GAL4 had a slight, but significant effect on the average flight value (*FI* = 4.99 ± 0.09) whereas *UAS-Act57B*^*K328Q*^ and *UAS -Act57B*^*K326Q/K328Q*^ expression abolished IFM function (*FI* = 0.11 ± 0.02 and 0.08 ± 0.04, respectively). Notably, Mef2-GAL4-driven *UAS-Act57B*^*K326Q/K328Q*^ actin expression predominantly resulted in pupal lethality with relatively few adult flies emerging from their puparia. Thus, Mef2-GAL4-driven GFP-tagged or acetyl-mimetic Act57B actin impaired flight and muscle performance.

To assess if expression of acetyl-mimetic cardiac actin affected additional somatic musculature, the climbing ability of *w*^*1118*^, *Mef2-GAL4*> *UAS-Act57B*^*WT*^, *UAS-Act57B*^*K326Q*^, *UAS-Act57B*^*K328Q*^, and *UAS-Act57B*^*K326Q/K328Q*^ flies was examined (Figure 6B). Expression of *UAS-Act57B*^*WT*^ actin had no significant effect on climbing distance (8.33 ± 0.54 cm) relative to *w*^*1118*^ controls (8.85 ± 0.49 cm). All acetyl-mimetic actin-expressing flies, however, exhibited climbing defects compared to *w*^*1118*^ and to *Mef2-GAL4*> *UAS-Act57B*^*WT*^ *Drosophila*. Flies expressing *UAS-Act57B*^*K326Q*^ had a small but significant reduction in climbing ability (6.14 ± 0.50 cm), while flies expressing *UAS-Act57B*^*K328Q*^ or *UAS-Act57B*^*K326Q/K328Q*^ had strikingly reduced climbing capabilities (4.22 ± 0.30 cm and 0.93 ± 0.24 cm respectively) compared to controls. Furthermore, flies expressing *UAS-Act57B*^*K326Q/K328Q*^ actin performed significantly worse than those expressing *UAS-Act57B*^*K328Q*^ actin.

### *Mef2-GAL4*> *UAS-Act57B*^*K328Q*^ and *UAS-Act57B*^*K326Q/K328Q*^ lack of flight is associated with loss of IFM fibers

In addition to inducing a lack of flight, Mef2-GAL4> *Act57B*^*K*328*Q*^ and *Act57B*^*K*326*Q*/*K*328*Q*^ acetyl-mimetic cardiac actin expression also resulted in a “wings up” phenotype. This phenotype is commonly associated with “hypercontracted” and damaged IFM that occurs due to mutations in *Drosophila* muscle proteins (Fyrberg et al., 1990; Beall and Fyrberg, 1991; Kronert et al., 1995; An and Mogami, 1996; Reedy et al., 2000; Nongthomba et al., 2003). Polarized light microscopy was employed to investigate disturbances in gross IFM morphology (Figure 6C). *Mef2-GAL4*> *UAS-Act57B*^*WT*^ and *UAS-Act57B*^*K326Q*^ *Drosophila* displayed similar IFM fiber structure. Both perpendicularly-oriented sets of fibers were continuous and straight, spanning the entire thorax in each line. However, *Mef2-GAL4*> *UAS-Act57B*^*K328Q*^ and *UAS-Act57B*^*K326Q/K328Q*^ *Drosophila* were characterized by the absence of continuous IFM fibers. Only occasionally was light observed emerging from birefringent material closely associated with the thoracic cuticle, which we assumed were IFM fiber remnants. Interestingly, *Mef2-GAL4*> *UAS-Act57B*^*K328Q*^; *Mhc*^*10*^/+ *Drosophila* displayed a greater abundance of thoracic material and birefringent musculature consistent with previous studies that demonstrated a reduced number of myosin motors can suppress mutant fiber destruction (Beall and Fyrberg, 1991; Nongthomba et al., 2003). However, the extent of suppression was incomplete as IFMs were still largely absent.

### Relative to *UH3-GAL4, Act88F-GAL4* drives elevated expression levels of IFM-restricted transgenic actin

**Figure 7:**
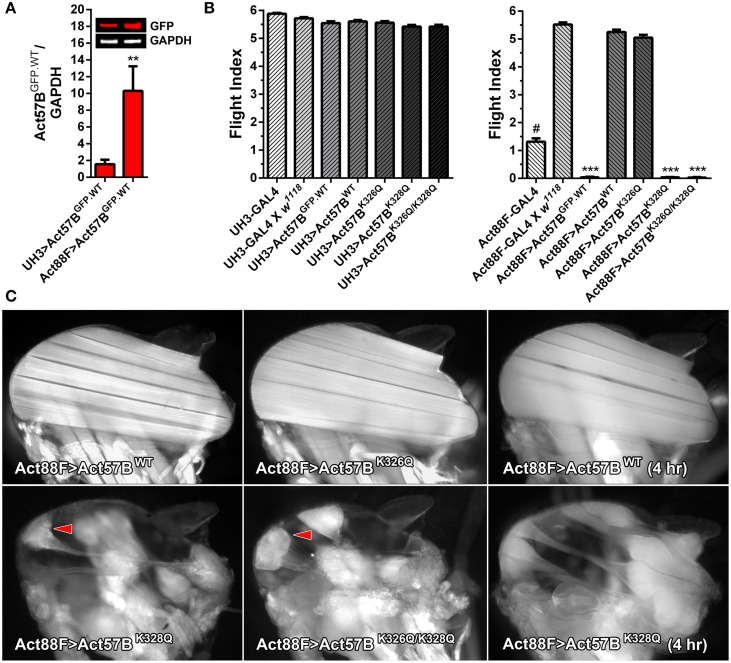
**Disproportionately high expression of *UAS-Act57B*^*K328Q*^ via the Act88F-GAL4 IFM-specific driver induces hypercontraction. (A)** Quantitative western blot analysis of Act57B^GFP.WT^ abundance driven by UH3-GAL4 vs. Act88F-GAL4. GFP intensities were normalized to that of GAPDH and averaged for eight IFM samples with four technical replicates each. Act88F-GAL4 drove significantly higher amounts of transgenic actin relative to UH3-GAL4 (^**^P < 0.01). **(B)** Flight indices of *UH3-GAL4*> and *Act88F-GAL4*> *UAS-Act57B*^*GFP.WT*^, *UAS-Act57B*^*WT*^, *UAS-Act57B*^*K326Q*^, *UAS-Act57B*^*K328Q*^, and *UAS-Act57B*^*K326Q/K328Q*^ transgenic *Drosophila*. UH3-GAL4, and “low dose” expression of all *UAS-Act57B* actin constructs by the driver, had no effect on flight. *Act88F-GAL4 Drosophila* exhibited significantly reduced flight performance relative to female progeny of *Act88F-GAL4* x *w*^*1118*^ (^#^*P* < 0.001). The latter demonstrated wild-type-like flight ability. “High-dose” expression of *UAS-Act57B*^*GFP.WT*^ by Act88F-GAL4 eliminated flight whereas *UAS-Act57B*^*WT*^ transgene expression had no effect on flight performance. “High dose” expression of *UAS-Act57B*^*K326Q*^ reduced flight ability, an effect which approached statistical significance. *Act88F-GAL4*> *UAS-Act57B*^*K328Q*^ and *UAS-Act57B*^*K326Q/K328Q*^ *Drosophila* were flightless (^***^P < 0.001). **(C)** Fluorescent images of dorsal longitudinal IFMs from two-day-old *Act88F-GAL4*> *UAS-Act57B*^*WT*^, *UAS-Act57B*^*K326Q*^, *UAS-Act57B*^*K328Q*^, and *UAS-Act57B*^*K326Q/K328Q*^ flies. *Act88F-GAL4*> *UAS-Act57B*^*K326Q*^ IFMs were indistinguishable from *Act88F-GAL4*> *UAS-Act57B*^*WT*^ control IFMs. *Act88F-GAL4*> *UAS-Act57B*^*K328Q*^ and *UAS-Act57B*^*K326Q*/*K328Q*^ *Drosophila*, however, displayed hypercontracted IFM with separated and bunched fibers (red arrowheads) at attachment sites. IFMs from young (four hour old) *UAS-Act57B*^*K328Q*^-expressing flies had a considerably less severe phenotype with minimal thinning and separation of the fibers.

IFM-specific GAL4 drivers provide an additional resource to further characterize the effects of pseudo-acetylated cardiac actin on flight muscle function and morphology. To quantify differences in transgenic protein abundance exclusively in IFMs, dissected fibers from flies expressing *Act57B*^*GFP.WT*^ driven by either UH3- or Act88F-GAL4, as well as fibers from control “non-driven” flies, were subjected to quantitative western blot analysis (Figure 7A). The signal intensities from the GFP and actin bands were measured and normalized to that from GAPDH. As found in whole thoraces, GFP signals in IFMs from all control lines were negligible (not shown), while IFMs from *UH3*- and *Act88F-GAL4*> *UAS-Act57B^GFP.WT^* flies showed detectable amounts of GFP-actin. Act88F-GAL4 induced significantly higher expression levels of GFP-actin in the IFMs compared to UH3-GAL4 (10.31 ± 2.94 vs. 1.57 ± 0.52). No differences in the relative abundance of non GFP-labeled, endogenous IFM actin were identified (not shown).

### IFM-specific expression of GFP-labeled or acetyl-mimetic actin decreases flight ability in a dose-dependent manner

Flight tests were performed on two-day-old *UH3*- and *Act88F-GAL4* driver lines and on the progeny of each driver line crossed to *w*^*1118*^ control, *UAS-Act57B*^*GFP.WT*^, *UAS-Act57B*^*WT*^, *UAS-Act57B*^*K326Q*^, *UAS-Act57B*^*K328Q*^, or *UAS-Act57B*^*K326Q/K328Q*^ transgenic *Drosophila* (Figure 7B). The *UH3-GAL4* line, and the progeny of *UH3-GAL4* crossed to *w*^*1118*^ displayed unperturbed flight ability (*FI* = 5.88 ± 0.03 and 5.72 ± 0.05, respectively). “Low dose” expression of all *UAS-Act57B* actin constructs using the UH3-GAL4 driver had no effect on flight ability. All lines displayed flight indices similar to controls (*FI* = 5.42 – 5.61). Therefore, UH3-GAL4-driven GFP-tagged, wildtype, or acetyl-mimetic Act57B permitted flight.

*Act88F-GAL4* (88F2) *Drosophila* displayed markedly reduced flight ability (*FI* = 1.31 ± 0.12) (Figure 7B). This was consistent with impaired flight phenotypes observed with publicly available *Act88F-GAL4* lines (w^*^; *P{Act88F-GAL4.1.3}3* and w^*^; *P{Act88FGAL4.1.3}81B*, *P{Act88F:GFP}2/ SM6b*) (not shown). When crossed to *w*^*1118*^ control flies however, female progeny harboring a single *Act88F-GAL4* (88F2) gene exhibited wildtype-like flight performance (*FI* = 5.52 ± 0.07). In contrast, no progeny from the two publicly available *Act88F-GAL4* lines, when crossed to *w*^*1118*^ control flies, regained flight ability (not shown). Therefore, we exclusively tested and compared flight performance of the female offspring of *Act88F-GAL4* (88F2) *Drosophila* crossed to each *UAS-Act57B* actin transgenic line.

“High dose” *Act88F-GAL4*> *UAS-Act57B^GFP.WT^* expression eradicated flight (*FI* = 0.04 ± 0.02) (Figure 7B). Notably, *Act88F-GAL4*> *UAS-Act57B^WT^* flies demonstrated wildtype-like flight ability (*FI* = 5.25 ± 0.09). As with Mef2-GAL4, expression of *UAS-Act57B*^*K326Q*^ via Act88F-GAL4 slightly depressed flight ability, which closely approached statistical significance. However, Act88F-GAL4-mediated expression of *UAS-Act57B*^*K328Q*^ and *UAS-Act57B*^*K326Q/K328Q*^ caused a complete loss of flight (*FI* = 0.04 ± 0.01 and 0.03 ± 0.01, respectively).

### The *Act88F-GAL4*> *UAS-Act57B*^*K328Q*^ and *UAS-Act57B*^*K326Q/K328Q*^ flightless phenotype is associated with hypercontracted IFM

Fluorescent microscopy was employed to inspect the IFM histopathology in the acetyl-mimetic relative to control flies (Figure 7C). Two-day-old *Act88F-GAL4*> *UAS-Act57B*^*K326Q*^ *Drosophila* did not show obvious differences in fiber morphology compared to *Act88F-GAL4*> *UAS-Act57B*^*WT*^. Similarly-aged *Act88F-GAL4*> *UAS-Act57B*^*K328Q*^ and *UAS-Act57B*^*K326Q/K328Q*^ *Drosophila*, in contrast, showed prominently hypercontracted IFM fibers, characterized by separation and accumulation of fiber material at one or both attachment sites as previously observed in other *Drosophila* muscle mutants (Fyrberg et al., 1990; Nongthomba et al., 2003). IFMs in very young *UAS-Act57B*^*K328Q*^ expressing adults (< four hour old) had a substantially less severe phenotype (Figure 7C). Most fibers could be distinctly visualized with minimal thinning and separation. The results indicated that the hypercontracted phenotype associated with Act57B^K328Q^, and potentially with Act57B^K326Q/K328Q^ acetyl-mimetic actin, was progressive and deteriorates with age and/or use.

## Discussion

Post-translational modifications represent a means to reversibly or irreversibly alter the physical and chemical nature of proteins. PTMs thereby modulate the molecules' properties and dramatically increase the complexity of biological systems. For example, even a single protein can exist as a diverse mixture of many modified forms (Agnetti et al., 2011). Specific measurements of a number of PTMs have revealed that several residues of a particular protein can be modified. Moreover, different PTMs may compete with each other for access to a single residue on the same protein. Based on an annotated human database, 62% of cardiac proteins have at least one PTM with phosphorylation dominating, whereas 25% have multiple types of modifications (van Eyk, 2011).

The cardiac thin filament is subject to a host of PTMs that markedly influence the properties of the constituent subunits and can directly affect contractile regulation and muscle performance (Metzger and Westfall, 2004; Sumandea et al., 2004; Agnetti et al., 2011; Solaro and Kobayashi, 2011; van Eyk, 2011). However, the modified status of these proteins is infrequently accounted for in *in silico* and *in vitro* experiments. As investigation and discovery of myocardial protein PTMs intensify, determining the *in vivo* consequences of modifying amino acid residues that lie at highly conserved and at potentially critical locations becomes increasingly important. Establishing model systems that limit genetic diversity and benefit from robust and relatively efficient transgenic tools for organism development and high throughput physiological assessment is imperative.

Here we provide novel data pertaining to recently identified PTMs using *Drosophila* that express pseudo-acetylated K326Q, K328Q, or K326Q/K328Q cardiac actin in a muscle-restricted manner. Transgenic actin expression was confirmed via thoracic and IFM-specific cDNA analysis and by GFP-based reporter imaging. As previously found with similar GFP-tagged constructs (Röper et al., 2005; Perkins and Tanentzapf, 2014), we observed restricted and repetitive incorporation of transgenic GFP-actin along IFM myofibrils, which, as evaluated by phalloidin labeling, were indistinguishable from wild-type myofibrils (not shown). Röper et al. reported that the IFM was the only muscle in which function was affected by overexpression of GFP-labeled actin (Röper et al., 2005). Muscle-restricted expression of muscle or cytoplasmic GFP-actins using the UAS-GAL4 system was shown to yield flightless adults that were otherwise healthy and fertile. Consistent with earlier studies, it was postulated that impaired IFM function, and in some cases the disrupted flight muscle structure, resulted from an imbalance in the relative amounts of actin and myosin (Beall et al., 1989; Bernstein et al., 1993; Röper et al., 2005; Vigoreaux, 2006). Furthermore, Fyrberg et al. (1998) showed that *Drosophila* exclusively expressing chimeric actin, consisting of part of Act57B fused with the remaining portion of Act88F in their IFM, exhibited decreased flight ability. Thus, in addition to actin and myosin stoichiometric discrepancies that potentially influence performance, these findings suggest functional non-equivalence of actin isoforms and that the IFM is also exquisitely sensitive to actin sequence variation. However, we observed that “low dose” expression of GFP-actin using the *MHC*- or *UH3-GAL4* driver did not impair flight and that expression of non-GFP-tagged wildtype Act57B actin via *MHC*-, *Mef2*-, *UH3*-, or *Act88F-GAL4* muscle-specific drivers had no influence on *Drosophila* flight ability. Therefore, despite differences in 9 amino acid residues between Act88F and Act57B actin isoforms (Figure 2), incorporation of non-mutant cardiac actin into IFM myofibrils appeared to support flight at expression levels dictated by each GAL4 driver. These results imply that the GFP moiety of excessively overexpressed GFP-fused actins directly impairs flight. This may be due, in part, to perturbed Tm movement or myosin crossbridge binding in the IFM consistent with the N-terminal fluorescent protein tag located proximal to Tm and myosin binding sites on actin. Importantly, our findings illustrate that GAL4-mediated transgene expression *can be employed to investigate the effects of non-GFP tagged cardiac actin* modifications on the readily measurable index of flight.

Compared to *UAS-Act57B*^*WT*^ cardiac actin expression, expression of mutant acetyl-mimetic cardiac actin had a dose-dependent effect on flight ability. Moreover, robust expression of *UAS-Act57B*^*K328Q*^ and *UAS-Act57B*^*K326Q/K328Q*^ via Mef2- or Act88F-GAL4 induced a greater reduction in flight performance relative to *UAS-Act57B*^*K326Q*^. Mef2- or Act88F-GAL4-driven Act57B^K326Q^ also had no resolvable effect on gross IFM morphology. It is conceivable that, rather than a direct effect of K→Q substitution on contraction, the milder phenotype associated with Act57B^K326Q^ might be attributable to reduced actin monomer incorporation, owing to a decrease in protein stability or increase in protease accessibility caused by the mutation. Though difficult to rule out confounding effects on protein stability, the preponderance of buttressing evidence suggests that Act57B^K326Q^ exerts direct yet modest effects on contractile function. Namely, in addition to slightly depressing flight ability, *Mef2-GAL4*> *UAS-Act57B*^*K326Q*^ flies exhibited significantly reduced climbing ability. Moreover, *Mef2-GAL4*> *UAS-Act57B*^*K326Q/K328Q*^ flies displayed impaired climbing relative to *Mef2-GAL4*> *UAS-Act57B*^*K328Q*^ flies. The latter results also highlight an influence of pseudo-acetylated actin on non-fibrillar adult somatic muscles.

The absence of flight in *Act57B*^*K328Q*^ and *Act57B*^*K326Q/K328Q*^-expressing flies was associated with a “wings up” phenotype and a loss of IFM fibers. Mutations in *Drosophila* muscle proteins frequently produce a phenotype referred to as hypercontraction (Fyrberg et al., 1990; Beall and Fyrberg, 1991; Kronert et al., 1995; An and Mogami, 1996; Reedy et al., 2000; Nongthomba et al., 2003). This degenerative syndrome is characterized by muscles that begin to develop normally, and then auto-destruct in an apparently myosin-dependent manner. The IFM of the troponin I and T mutants, *hdp*^*2*^ and *up*^*101*^, respectively, initially develop normally and begin to show signs of degeneration 78 h after puparium formation, concomitant with initial muscle twitching in the developing imago (Naimi et al., 2001; Nongthomba et al., 2003). By the second day of adult life very few sarcomeres remain (Beall and Fyrberg, 1991). The onset of hypercontraction suggests that it is a result of muscle activation, and is not due to abnormal development (Nongthomba et al., 2003).

Both *hdp*^*2*^ and *up*^*101*^ thin filaments exhibit aberrantly positioned Tm in the absence of Ca^2+^, which results in exposed myosin binding sites at rest (Cammarato et al., 2004; Viswanathan et al., 2014). Consequently, IFM hypercontraction is believed to result from excessive actomyosin interaction and unregulated force production. Models of the F-actin-Tm interface reveal K326 and K328 of actin participate in vital intermolecular electrostatic associations with Tm to establish an energetically favorable conformation (Brown and Cohen, 2005; Li et al., 2011; Barua et al., 2012, 2013; Lehman et al., 2013). Here, Tm is located in an azimuthal location that occludes myosin binding sites on F-actin. Moreover, a recent model of the F-actin-Tm-myosin interface reveals K328 on actin can also directly interact with strongly bound myosin heads (Behrmann et al., 2012). Our data suggest that sufficiently high acetylation of these actin residues can weaken actin-Tm interaction and disrupt the ability of Tm to properly block crossbridge formation and force transmission to the thin filament. Therefore, as with the aforementioned troponin mutations, the acetyl-mimetic actin may similarly trigger hypercontraction. Interestingly, the effect of modifying K328 triggered more severe defects relative to K326, which indicates K328 may be most essential for proper relaxation, *in vivo*. Moreover, since K328Q actin may also impair strong S1 binding, which is predicted to oppose hypercontraction, our data imply the effects of potentially weakening S1-actin association are less harmful than those that influence Tm positioning, as the muscles still hypercontract.

The importance of these actin residues in thin filament regulation, and the effects associated with potential loss of charge, are further underscored by naturally occurring mutations. For example, the K326N nemaline myopathy actin mutation, which differs from the K326Q acetyl-mimetic isoform investigated here by a single methylene bridge, was reported in patients with stiff muscles and spontaneous contractures, suggesting a hypercontractile phenotype (Jain et al., 2012). Computation of the energy landscape for this mutant revealed reduced actin-Tm interaction energy, which would facilitate a shift of Tm away from myosin binding sites, and would explain the increased Ca^2+^-sensitivity and hypercontractility of affected muscles (Jain et al., 2012; Orzechowski et al., 2014). Considering the severity of the effects accompanied by high expression of the K328Q mutation observed in the current study, similar charge loss at K328 may not be well-tolerated in higher organisms.

Based on F-actin-Tm, F-actin-Tm-myosin models, and our physiological data, we believe that acetylation of K326 and K328 of actin alters electrostatic associations with Tm and/or myosin, destabilizes Tm's inhibitory position, and thereby enhances actomyosin associations and promotes IFM hypercontraction and muscle destruction. However, our approach does not preclude possible alternative contributors to muscle pathology. For example, the amino acid substitutions may compromise the folding efficiency, thermal stability, and/or polymerization properties of actin filaments as recently observed for particular cardiomyopathy-causing lesions (Mundia et al., 2012; Müller et al., 2012). Thus, increased F-actin and thin filament lability may promote myofilament and sarcomeric degeneration. Additionally, though our data suggest that sufficiently high doses of actyl-mimetic actins are required to elicit dysfunction, we cannot rule out a potential contribution from early transgene activation via the Mef2-GAL4 driver, relative to the others, that disrupts muscle development (Markstein et al., 2008). Thus, the most severe IFM phenotype, which was observed in *Mef2-GAL4*> *UAS-Act57B*^*K328Q*^ and *UAS-Act57B*^*K326Q/K328Q*^ *Drosophila*, may be due to both abundant quantities and premature activation times of transgene expression. If K326 and K328 of actin are required for proper thin filament regulation, excessive myosin binding and force production during early muscle development may alter the core building blocks required for proper IFM formation and irreversibly disrupt myofibrillogenesis. This is not unreasonable since during development actomyosin associations appear compulsory for well-ordered and properly functioning IFM (Cripps et al., 1999). However, we detected a greater abundance of birefringent IFM material in *Mef2-GAL4*> *UAS-Act57B*^*K328Q*^ thoraces when myosin was reduced. Moreover, relative to *Mef2-GAL4*> *UAS-Act57B*^*K328Q*^, delayed expression of *UAS-Act57B*^*K328Q*^ actin via Act88F-GAL4 led to a less severe phenotype characterized by post-eclosion progressive separation and accumulation of fiber material to IFM attachment sites. Thus, we interpret these results as consistent with the previously described myosin-dependent, degenerative hypercontraction syndrome and not with a complete lack of IFM development (Nongthomba et al., 2003).

Here we provide *in vivo* confirmation of a requirement for positively charged lysine residues at amino acid positions 326 and 328 on actin for proper thin filament function. We demonstrate how PTMs that sufficiently mask these vital charges can have dramatic consequences on muscle performance and structure. While LC-MS/MS analysis of myofilament enriched subfractions of guinea pig hearts revealed lysines 326 and 328 were acetylated, stoichiometry was not assessed (Foster et al., 2013). Our current data suggest Mef2-GAL4-driven transgenic actin comprises approximately 10–20% of total actin (not shown), while MHC-GAL4 drives significantly less. Therefore, minor changes in a potentially small acetylated myofilamentous actin pool may have substantial repercussions on muscle properties. Masking the charges at K326 and K328 apparently increases muscle function by potentially lowering actin-Tm interaction energy, altering Tm positioning, and perpetually promoting myosin crossbridge formation and contraction. Disproportionately excessive amounts of K326 and K328 acetylation and hypercontractile activity in vertebrate hearts may be deleterious as observed here. Nonetheless, small populations of acetylated K326 and K328 could have beneficial effects under normal conditions that act to augment the contractile properties of muscle. In disease however, particularly afflictions characterized by nutrient excess such as diabetes, elevated acetyl-CoA levels may lead to increased acetylation of these critical actin residues and possibly exacerbate pathology.

Investigating the effects of myofilament protein modifications on distinct *Drosophila* muscles will facilitate our effort to understand the molecular basis of contractile regulation and, importantly, of potentially tempering myopathic responses. Biochemical, biophysical, and structural studies frequently neglect to account for PTMs of myofilamentous proteins, which can greatly modulate contractile behavior. Thus, to truly comprehend muscle performance in health and disease, consideration needs to be given to these dynamic protein modifications. *Drosophila* facilitates genetic manipulation of thin filament components and evaluation of the consequences of perturbation. Moreover, since abundant quantities of native IFM thin filaments and actin can be isolated for *in vitro* studies (Bing et al., 1998; Razzaq et al., 1999; Cammarato et al., 2004; Vikhorev et al., 2010), the models permit hierarchical investigation of the effects of such PTMs on contractile machinery from the molecular through the tissue level. Overall, our findings emphasize the utility of *Drosophila* as a model system that allows for control of genetic modifiers and environmental factors and that enables efficient targeted design and assessment of molecular and tissue-specific responses to protein modifications in the physiological context of muscle.

### Conflict of interest statement

The authors declare that the research was conducted in the absence of any commercial or financial relationships that could be construed as a potential conflict of interest.
